# Vitamin B12 Deficiency in Patients with Diabetes on Metformin: Arab Countries

**DOI:** 10.3390/nu14102046

**Published:** 2022-05-13

**Authors:** Jwaher Haji Alhaji

**Affiliations:** Department of Health Sciences, College of Applied Studies and Community Service, King Saud University, P.O. Box 145111, Riyadh ZIP 4545, Saudi Arabia; jalhejjy@ksu.edu.sa

**Keywords:** diabetes, metformin, vitamin B12, T2DM, Arab countries

## Abstract

Background: Diabetes is a global pandemic, especially in Arab countries. Aim: The goal of this study was to review the published studies that were conducted to determine the relationship between metformin treatment for type 2 diabetes mellitus (T2DM) and vitamin B12 deficiency and to identify possible complications in this relationship. Methods: I searched for all relevant studies published in English before 2020 on the PubMed and Web of Knowledge databases using the following terms: “metformin”, “vitamin B12”, “neuropathy”, “diabetes mellitus”, and Middle Eastern countries. Results: Eleven studies were included in this review which indicated an association between taking metformin and B12 deficiency in patients with T2DM in Arab countries. This B12 deficiency was found to be negatively associated with the dose and duration of metformin therapy. The physician’s knowledge of current ADA recommendations regarding supplementation with and screening of the B12 level for T2DM patients on metformin was also found to have an effect. Conclusion: Metformin therapy is associated with B12 deficiency among people with T2DM in Arabic countries. Thus, diabetes must be managed in compliance with current guidelines and recommendations, and B12 levels must be routinely monitored, particularly in those who have been long-term takers of metformin, to ensure the suitable management of diabetes and its complications.

## 1. Introduction

Diabetes is a serious and growing global health concern, according to the latest statistics. The global prevalence of impaired glucose tolerance was estimated at 7.5% (374 million) in 2019 and is projected to reach 8.0% (454 million) by 2030 and 8.6% (548 million) by 2045. The number of people with diabetes is anticipated to increase to 693 million by 2045 [1]. Most people with diabetes live in low- and middle-income countries, at approximately 79%, where a rapid increase in the number of diabetic cases is expected over the next 22 years by 13.3% and 13.9% in the Middle East and North Africa (MENA) Region [1,2]. Diabetes can be classified into two main categories: (1) insulin-dependent diabetes mellitus (T1DM), or type 1 diabetes mellitus, characterized by the lack of insulin secretion (without daily administration of insulin, T1DM rapidly becomes fatal); and (2) non-insulin-dependent diabetes mellitus, or type 1 diabetes mellitus (T2DM) [3]. T2DM results from the body’s ineffective use of insulin. About 90% of people with diabetes worldwide have T2DM, which is mainly associated with high body weight and physical inactivity (WHO, 2019) [1].

All guidelines, including the European Association for the Study of Diabetes (EASD) and the American Diabetes Association (ADA), consider metformin (1,1-dimethylpiguanide hydrochloride) a cornerstone and first-line treatment, along with lifestyle intervention, for managing hyperglycemia in patients with T2DM [4]. Furthermore, metformin has beneficial effects on carbohydrate metabolism, weight loss, and prevention of vascular disease [5].

Metformin is used as a monotherapy or in combination with other medicines [6]. However, one of its side effects is the reduction in vitamin B12 levels. Vitamin B12 deficiency is both underdiagnosed and undertreated [7,8]. Severe deficiency (e.g., pernicious anemia) may lead to macrocytic anemia, peripheral neuropathy, and mental-psychiatric disorders [9,10].

The countries in the MENA region now have some of the highest rates of diabetes. This high rate is due to a combination of factors including rapid economic development and urbanization, which have resulted in decreased levels of physical activity, high intakes of refined carbohydrates, and increased prevalence of obesity. An ageing population has resulted in a rapidly increased prevalence as age increases the risk of T2DM [3].

The MENA region countries have the second highest rate of increase in diabetes cases worldwide, with the number of people with diabetes projected to increase by 96.2% by 2035 [11]. Therefore, researchers have begun focusing on the effect of metformin use on the proportion of patients with vitamin B12 deficiency in this region in patients with T2DM. Therefore, in this study, I aimed to conduct a preliminary review of the literature (observational and interventional studies) to determine the relationship between metformin use for treatment of T2DM and vitamin B12 deficiency (as measured by vitamin B12 concentration in the blood), and to identify possible complications.

## 2. Materials and Methods

### 2.1. Literature Selection and Eligibility Criteria

**Figure 1:**
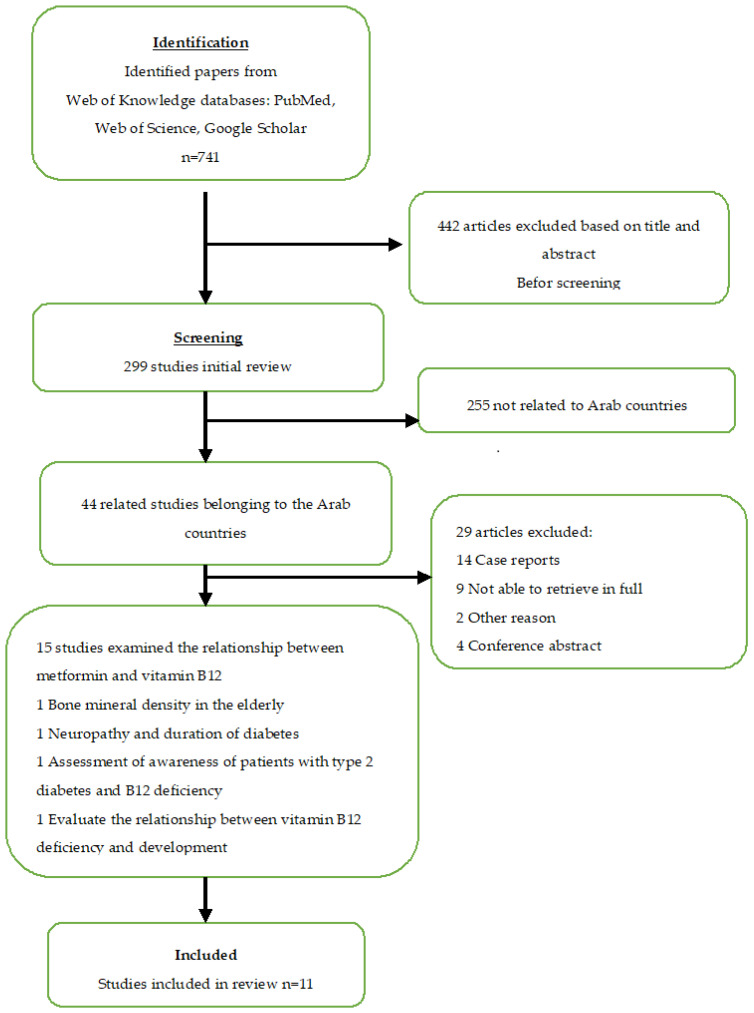
Research scheme and exclusion criteria.

I searched for all relevant studies published in English prior to 2020 on Web of Knowledge databases: PubMed, Web of Science, and Google Scholar, using the following search terms: “metformin”, “vitamin B12”, “neuropathy”, “diabetes”, and Middle Eastern countries. I examined the reference lists of specific studies as well as those in relevant reviews to identify eligible studies that were not identified through the initial search. I then conducted a selective review of the literature, studies, and specific reviews in Arab countries that investigated the association of metformin use with vitamin B12 deficiency in T2DM patients. Regarding the treatment, I identified 44 articles, and selected those focusing on metformin, vitamin B12, neuropathy, type 2 diabetes, and Arab countries. The final number of studies was 15; I also selected 11 related cognitive studies, including 1 study that dealt with the level of awareness of patients and physicians, as shown in Figure 1.

### 2.2. Inclusion and Exclusion Criteria

Studies considered eligible for the meta-analysis were comparative studies published in English comparing either vitamin B12, neuropathy, or anemia in patients with diabetes using metformin with no metformin users, or in patients with diabetes and a history vs. no history of metformin use. Eligible studies also had to provide data on at least one of the following outcome measures: incidence of vitamin B12 deficiency, neuropathy or anemia, serum vitamin B12 concentration, and percentage change in serum vitamin B12 concentrations from baseline. I reviewed the titles and abstracts of studies obtained through the initial keyword search to identify potentially eligible articles; then, I examined the full text of these articles to obtain a definitive list of included studies.

## 3. Results

From the databases, I identified 7257 records; finally, 11 studies were included in this review [12,13,14,15,16,17,18,19,20,21,22]. Figure 1 shows the results of the screening and selection process. The full results of the search are shown in Table 1. Of the 11 scientific papers, 4 were conducted in the Kingdom of Saudi Arabia [16,18,19,21], 2 in Palestine [14,22], and 1 each in Libya [12], Lebanon [13], Qatar [15], Oman [20], and the United Arab Emirates (UAE) [17]. To the best of my knowledge, with this review, I am the first to explore the association between metformin use and vitamin B12 deficiency in individuals with T2DM in Arab countries. Most studies included in this review reported significantly lower vitamin B12 levels in patients administered metformin. Observational data showed lower vitamin B12 levels and an increased risk of borderline or vitamin B12 deficiency with metformin use. The seven studies in Table 1 indicated either an association of metformin use with a lower vitamin B12 concentration [13,14,16,17,19,20,21] that was highly significant (*p* ≤ 0.01) or was not significantly different (432 ± 206 vs. 448 ± 219 pg/ml; *p* = 0.4 in one study [12]. In another study, a slight decrease was found [15]. Additionally, several studies indicated an inverse relationship between the duration of metformin use and the serum concentration of vitamin B12 in patients with T2DM [13,14,16,17,19,22]. A significant difference was reported between metformin dose and serum concentration of vitamin B12 [14,16,18,19,20,21].

Initially, the reference studies cross-sectional in design. In eight studies, the participants included both sexes. The Libya study indicated that men had significantly higher levels of vitamin B12 deficiency than women (512 ± 226 vs. 399 ± 193; *p* = 0.001). Libya and Qatar patients with T2DM on metformin had a lower prevalence of vitamin B12 deficiency [12,15]. However, studies that found a higher prevalence of vitamin B12 deficiency in metformin users than in no metformin users reported a statistically significant association (*p* ≤ 0.001) [16,17,19]. The duration and dose of metformin were observed to be closely related to vitamin B12 deficiency. A connection was found between vitamin B12 deficiency and metformin dose. The vitamin B12 deficiency group used a significantly higher daily dose of metformin (*p* < 0.05) [16,19,20,21], as was also reported (*p* < 0.001) in the Palestine study [14]. Among the studies that found a relationship between duration of metformin intake and vitamin B12 deficiency, the minimum and maximum durations reported by the studies were 3 months [13,14] and 10 years, respectively [15]. The yearly increase in the duration of diabetes was associated with an 18.6 ± 6.7 pg/mL (*p* = 0.007) lower vitamin B12 level [14]. The percentage of vitamin B12 deficiency in patients with T2DM who used metformin was 48% (*p* = 0.002) [14].

## 4. Discussion

Diabetes is a global problem, especially in Arab countries. Metformin, the standard first-line treatment for diabetes, raises the risk of vitamin B12 deficiency [14]. To the best of my knowledge, this is the first review in Arab countries of studies standardizing the effects of exposure to different levels of metformin on vitamin B12 levels in patients with T2DM.

Overall, 11 observational studies in this review reported lower levels of vitamin B12 and an increased risk of vitamin B12 deficiency with metformin administration to patients with T2DM [21,22]. Chapman et al., in a systematic review of 17 cross-sectional studies, found a connection between lower serum levels of vitamin B12 and metformin intake [23]. The studies’ results described a normal vitamin B12 concentration (>220 pg/mL), potential vitamin B12 deficiency (150–220 pg/mL), and apparent vitamin B12 deficiency (<150 pg/mL) [24]. However, researchers reported a predominance of vitamin B12 deficiency of 48% in metformin users in the UAE [17], which is a high percentage compared with those reported by other researchers that determined the prevalence of vitamin B12 deficiency among patients with diabetes, including a prevalence of 7% in the U.S. [25], 28.1% in South Africa [26], 22.5% in Brazil [27], 18.7% in New Zealand [28], and 9.7% in the Netherlands [29]. These differences can be explained by other factors that can affect the serum vitamin B12 concentration in people with T2DM in Arab countries that were not addressed in these studies, such as diet, other drug interactions, and lifestyle. Ethnic background and race are important factors to consider when assessing vitamin B12 levels with metformin use. Therefore, dissimilarities in vitamin B12 metabolism, transport, and intracellular handling among different ethnicities may explain the differences in the results obtained by different studies [30,31].

The results of studies in Arab countries agree that an association exists between the duration of metformin intake and vitamin B12 deficiency. Ting et al. [32] hypothesized that for every 1 g/day increase in metformin dose, the general risk of developing vitamin B12 deficiency increases by 2.88% (95% CI, 2.15–3.87). Although most studies related vitamin B12 deficiency with extended use of metformin, a randomized study clarified that in just 16 weeks of metformin use, serum folate and vitamin B12 levels decreased by 7% and 14%, respectively, in patients with T2DM [33].

In this review, serum vitamin B12 levels were associated with not just the duration of treatment, but also the metformin dose in patients with T2DM [32]. Therefore, the metformin dose was the strongest independent indicator of vitamin B12 deficiency, as evidenced by results that indicated a high level of significance in the vitamin B12 deficiency group that used a significantly higher daily dose of metformin (*p* < 0.01 [16,19,20,21]; *p* < 0.001 [14]). These results agree with those of a study conducted in the Netherlands in which the dose, but not the duration, of metformin was associated with vitamin B12 deficiency [34]. In another study, serum vitamin B12 levels were not associated with metformin dose but were associated with the duration of treatment [32]. Both appear to be independent risk factors of vitamin B12 deficiency. This difference can be explained by the effect of the regularity of metformin use and determining the dose and duration of metformin use.

Other researchers concluded that the use of metformin in patients with T2DM is associated with vitamin B12 deficiency and worsening clinical neuropathy in a dose-dependent manner [35]. The 2018 ADA Clinical Practice Recommendations endorse the screening of metformin users for vitamin B12 deficiency, whereas the 2017 ADA diabetic neuropathy statement recommends that all patients with diabetic neuropathy be assessed for vitamin B12 deficiency to exclude a treatable cause of neuropathy [36]. No clear relationship of vitamin B12 deficiency was evident with either metformin therapy or neuropathy [10]. However, patients with T2DM with and without vitamin B12 deficiency had a similar prevalence and severity of sensory and traumatic neuropathy [15,22]. Neuropathy was induced because vitamin B12 deficiency precedes the onset of megaloblastic anemia [37].

One study showed that many clinicians are unaware of or are not updated on current ADA recommendations regarding vitamin B12 supplementation and screening in patients with T2DM on metformin [18]. The lower prescription rates for vitamin B12 or B complex supplements (12.6%) can be attributed to a lack of knowledge regarding the association of metformin with vitamin B12 deficiency or the inconsistent international guidelines [12]. Routine tests for serum vitamin B12 level are not widely performed in patients with diabetes in most care centers in Arab countries. Therefore, clinicians need to be aware of the latest recommendations and must apply the current guidelines on the management of diabetes to avoid negative consequences.

## 5. Conclusions

In conclusion, the overall lower serum vitamin B12 levels in patients with T2DM are associated with a longer duration and higher dose of metformin use in Arab countries. Therefore, further studies must be conducted to identify patients who may benefit from vitamin B12 supplementation. Additionally, the routine checking of serum vitamin B12 levels is required in patients with T2DM, and clinicians should be provided with the ADA recommendations regarding the management of diabetes and its complications.

## Figures and Tables

**Table 1:** Summary of studies included in this review.

Authors/Year	Location	Study Duration	Study Sample Age (Years)	N	Duration of Metformin Intake	Daily Dose of Metformin (mg/Day)	Primary Outcome: B12 Deficiency
Elsaier et al., 2017 [12]	Libya	One year	Mean ± SD age 58.6 ± 9.9	500 patients receiving metformin M = 175 35% F = 325 65%	NA	NA	Mean B12 level = 463 ± 467 pg/mL, 63 (12.6%). Patients had a recent history of multivitamin or vitamin B complex intake. Vitamin B12 levels were <159 pg/mL in 2%, no significant difference in B12 serum levels between those who did and did not use metformin (432 ± 206 vs. 448 ± 219 pg/mL; *p* = 0.4)
Zalaket et al., 2018 [13]	Lebanon.	First 6 months of 2015	18–90	200 patients receiving metformin	>3 months *n* = 43 >12 months *n* = 157	NA	A highly significant difference between the B12 serum levels with the dose and duration of metformin intake. B12 deficiency levels decreased to 177.83 pmol/L with increasing duration of metformin use for those taking metformin for more than 12 months (*p* = 0.004) **.
Saqer et al., 2018 [14]	Palestine	NA	Mean ± SD age 54.48 ± 7.3	73 patients receiving metformin M = 38 52.05% F = 35 47.95%	Mean ± SD months 41.01 ± 26.4 <5 years *n* = 53 ≥5 years *n* = 20	Mean ± SD mg 1135.1 ± 476.4 <1000 *n* = 33 ≥1000 *n* = 40	Mean B12 level significantly lower in participants who used metformin for ≥5 years; 100 mg increase in metformin dose associated with a lowering of B12 level 19.3 ± 4.4 pg/mL (*p* ≤ 0.01) **
Elhadd et al., 2018 [15]	Qatar	6 March–28 September 2017	Mean ± SD age 54.19 ± 11.61	362 patients Receiving metformin = 235, non- metformin = 64	NA	NA	B12 levels not associated with use of metformin. B12 deficiency (B12 < 133 pmol/L, *p* < 0.01) ** was lower in metformin users (8%), whereas non-metformin users showed highly decreased levels of B12 (19%).
Alharbi et al., 2018 [16]	KSA	January–June 2016	57.8 ± 0.6 ^a^ 56.6 ± 1.4 ^b^	412 patients receiving metformin = 319, non- metformin = 93	<1 year *n* = 1 1–4 *n* = 123 >4 years *n* = 196	<1000 *n* = 6 (1.88) 1000–2000 *n* = 301 (94.36) >2000 *n* = 12 (3.76)	Metformin users had a significantly higher prevalence of vitamin B12 deficiency (9.4% vs. 2.2%, *p* < 0.036) *. Low levels of B12 occurred when metformin was taken at a dose > 2000 mg/day (AOR, 21.67; 95% CI, 2.87–163.47) or for >4 years (AOR, 6.35; 95% CI, 1.47–24.47).
Shahwan et al., 2018 [17]	UEA	June 2014–February 2015	Mean ± SD age 49.5 ± 7.8	200 patients receiving metformin M = 102 51% F = 98 49%	Mean ± SD years 3.35 ± 1.881	NA	Patients on metformin had statistically lower B12 levels (*p* = 0.002) **, of which metformin duration was the significant predictor (*p* < 0.0001) **.
Alshammari et al., 2019 [18]	KSU	Auguast–November 2018	Mean ± SD age 53.72 ± 11.31	363 patients receiving metformin M = 206 56.7% F = 157 43.3%	Mean ± SD years 9.84 ± 7.29	500 *n* = 2 (0.6%) 750 *n* = 205 (56.5%) 1000 *n* = 62 (17.1%) 1500 *n* = 93 (25.6%) 2000 *n* = 1 (0.3%)	12.5% of 16 patients had vitamin B12 levels below 200 pg/mL. Many physicians are not aware of the current ADA recommendations regarding vitamin B12.
Abdel Gawad 2019 [19]	KSA	December 2018–October 2019	48.22 ± 6.86 ^a^ 48.15 ± 6.79 ^b^	230 patients M = 180 F = 50	<4 years *n* = 10 (6.7%) >4 years *n* = 140 (93.3%)	Mean ± SD 1703.33 ± 261.30	Prevalence of B12 deficiency was higher in metformin users than in non-metformin users. Vitamin B12 deficiency and the dose and duration of metformin use were associated.
Al-Hamdi et al., 2020 [20]	Oman	January–December 2017	Mean ± SD age 55.3 ± 10.0	248 patients M = 98 (39.5) F = 150 (60.5)	<4 years *n* = 74 9.8% 4–10 *n* = 138 55.6% >10 *n* = 36 14.5%	<2000 *n* = 62 (25.0%) ≥2000 *n* = 186 (75.0%)	26 (10.5%) participants with T2DM had B12 deficiency with metformin treatment; borderline deficiency was found in 53 (21.4%) participants. A higher proportion of those receiving metformin doses ≥ 2000 mg had vitamin B12 deficiency (*p* = 0.004) **. No association between the duration of metformin use and B12 deficiency.
Jajah et al., 2020 [21]	KSA	March–May 2019	Mean ± SD age 58.10 ± 14.31	347 patients M = 136 F = 211	NA	500 *n* = 176 750 *n* = 117 1000 *n* = 54	10.4% prevalence of vitamin B12 deficiency among participants Deficient levels of B12 (<200) were more prevalent with metformin dose of 1 g and 750 mg than 500 mg; the association was statistically significant (*p* < 0.05) **.
Shahwan et al., 2020 [22]	Palestine	NA	Mean ± SD age 53.3 ± 9.7	400 patients M = 109 27.2% F = 291 72.8%	0–5 years *n* = 260 65.0% 5.1–10 years *n*= 120 30.0% 10.1–15 years *n*= 15 3.7% 15.1–20 years *n* = 5 1.3% >20 years *n* = 0 0.0	NA	A significantly negative association (*p* = 0.198) between low serum vitamin B12 level and metformin treatment; 38.4% of participants with low serum vitamin B12 were using metformin.

All data are reported as mean ± SD unless otherwise stated. NA: not applicable; T2DM: type 2 diabetes mellitus; M = male; F = female; KSU: Kingdom of Saudi Arabia; UAE: United Arab Emirates. a: metformin user; b: non-metformin user. * significant (*p* < 0.05), ** highly significant (*p* < 0.01).

## Data Availability

Not applicable.
